# The Interplay Between Chamber Musicians During Two Public Performances of the Same Piece: A Novel Methodology Using the Concept of “Flow”

**DOI:** 10.3389/fpsyg.2020.618227

**Published:** 2021-01-06

**Authors:** Eva Bojner Horwitz, László Harmat, Walter Osika, Töres Theorell

**Affiliations:** ^1^Department of Clinical Neuroscience, Karolinska Institutet, Stockholm, Sweden; ^2^Department of Music, Pedagogy and Society, Royal College of Music, Stockholm, Sweden; ^3^Department of Neurobiology, Care Sciences and Society, Center for Social Sustainability, Karolinska Institutet, Stockholm, Sweden; ^4^Department of Psychology, Faculty of Health and Life Sciences, Linnaeus University, Växjö, Sweden; ^5^Northern Stockholm Psychiatry, Stockholm, Sweden; ^6^Stress Research Institute, Stockholm, Sweden

**Keywords:** synchronization, musical performance, heart rate variability, flow, interpersonal interaction

## Abstract

The purpose of the study is to explore a new research methodology that will improve our understanding of “flow” through indicators of physiological and qualitative state. We examine indicators of “flow” experienced by musicians of a youth string quartet, two women (25, 29) and two men (23, 24). Electrocardiogram (ECG) equipment was used to record heart rate variability (HRV) data throughout the four movements in one and the same quartet performed during two concerts. Individual physiological indicators of flow were supplemented by assessments of group “state flow” (means from standardized questionnaires) and a group interview in which the musicians provided qualitative data. A matrix was constructed for the characterization of different kinds of demands in the written music in each one of the four movements for each one of the musicians. HRV derived from ECG data showed non-significant trends for group state flow across the eight musical episodes. Individual-level analysis showed that compared to the other players the first violin player had the highest mean heart rate and the lowest increase in high frequency (HF) power in HRV during this particular movement, particularly during the second concert. The qualitative data illustrated how an interplay of synchronized social interactions between this player and their colleagues during the musical performance was associated with a feeling of group state flow and served to support the first violinist. The case illustrates that the proposed mixed methodology drawing on physiological and qualitative data, has the potential to provide meaningful information about experiences of a flow state, both at individual and group levels. Applications in future research are possible.

## Introduction

We know from previous research that the interaction between individual musicians and their social and material environments becomes part of their performance. The interplay between music ensembles and their audiences becomes more dynamic when the musicians start to play (Bishop, 2018). The various components involved in a performance are interrelated and interdependent and are grounded in the specific social activity through which one perceives music. In our wider research, we want to improve our understanding of the experience of “flow,” a relaxed and at the same time intensely focused state. Flow is characterized by a sense of total immersion in an activity leading to a sense of control, lack of self-awareness, and enhanced performance (Csikszentmihalyi, 1990). We are simultaneously interested in the interactional synchronization experienced by groups and its relationship to flow. Part of this is to describe and understand the interplay among musicians during flow. In this paper, we illustrate our methodological contribution by examining a specific performance on two different occasions as an opportunity for evaluation. A novel set of measures are taken from these performances that combine physiological and qualitative data. We combine measurements of heart rate variability (HRV) with data collected *via* two scales: a shortened form of Flow State Scale (FSS-2) to assess flow experiences within a particular event (Jackson and Ekelund, 2004) – and the Flow Synchronization Questionnaire (FSyQ; Magyaródi and Oláh, 2015). A matrix was also constructed for the characterization of different kinds of demands in the written music. Our aim is to present a case study of possible methodologies that show how HRV, in combination with qualitative data provided by the scales, can be used as an indicator for a state of flow.

The study is set within *Musethica*, an international program which offers young chamber musicians advanced training. It aims to “*translate*” and bring to life musical text and musical information from paper to air and audience (musethica.org). One of the distinctive features of the program is that the musicians are given the opportunity to perform to audiences that may not be accustomed to classical music. The concerts in the program are free for the audience and take place in public and community spaces such as schools (school children), psychiatric departments (patients), factories (workers), and culture unions (migrants). In schools, the concerts are integrated into the school day with compulsory attendance and the pieces are played according to the full format in which the composer wrote them, rather than in an abbreviated version. Time is added after the concert for students to ask questions to the musicians. The children in the audience are not typically familiar with concert music of this type. The goal is to provide musicians with an opportunity to feel what it is like when a lay audience is touched by a performance; that is, fully engaged by the music. A supplementary goal of the program is to offer new audiences the chance to encounter classical music and to interact with young musicians.

Due to our interest in flow, we decided to collect data with a mixed set of parameters and took this opportunity to develop a novel methodology that might inform future research. We performed electrocardiographic (ECG) recordings for HRV in all four musicians for the duration of their performances. After each performance, the musicians filled in a short-standardized questionnaire regarding their individual experience of flow as well as experiences of synchronized social interactions, an indicator of group state flow. Finally, a qualitative interview was conducted with the group of musicians to inform and enhance the interpretation of the dataset. These measurements are explained in further detail in the following section.

## Flow and Heart Rate Variability

Our research takes an interest in how certain psychological states are transferred and shared among performers, in particular in the achievement of “flow” (Csikszentmihalyi, 1990). Flow is a form of optimal experience characterized as a subjective feeling wherein individuals are totally immersed in an activity; their attention becomes absorbed when challenges are in balance with skills (Csikszentmihalyi, 1990). Characteristics of the flow experience include a high degree of focus that feels effortless, a sense of control, loss of self-awareness, an altered experience of time, and enjoyment (Csikszentmihalyi and Nakamura, 2010).

It is believed that there is a connection between the autonomic nervous system (ANS) and the state of flow. Most studies of flow have indicated that there is an increased level of arousal observable in the individual, but interpretations can vary as to their explanation of how arousal states differ from experiences such as stress that would be considered as straining rather than enhancing (see review in Tozman and Peifer, 2016). Peifer et al. (2014) suggested that the relation of flow with sympathetic arousal follows an inverted U-curve rather than a linear function: moderate physiological arousal should facilitate flow-experience, whereas excessive physiological arousal should hinder flow.

Findings from previous studies have suggested that there is a nonreciprocal co-activation of the sympathetic and parasympathetic nervous system during flow experience (de Manzano et al., 2010; Harmat et al., 2015); and that therefore the flow experience is associated with increase in both parasympathetic and sympathetic arousal: a combination of relaxation and readiness for action. Such results support the notion that flow is a state of “effortless attention that can be experienced during task performance as a result of an interaction between emotional and attentional systems” (Ullén et al., 2010).

This response aligns with the function of the ANS, which is to adjust bodily systems in order to maintain or re-establish homeostasis when adapting to surrounding conditions (Cannon, 1935; Ulrich-Lai and Herman, 2009). Flow seems to necessitate the adaptive responses that the ANS performs; using sympathetic and parasympathetic systems. When exposed to stress, the body adjusts to the challenging situation in order to recover homeostasis. The parasympathetic branch and the sympathetic branch of the ANS respectively ensure adaptive adjustment at the physiological level (Cannon, 1935; Ulrich-Lai and Herman, 2009). During periods of acute stress, the sympathetic branch mobilizes energy reserves to cope with internal and external challenges. The stress response is characterized by changes such as increase in muscle tone and alterations of respiratory rate and heart rate (Cohen et al., 2007). The parasympathetic branch is related to the “rest and digest” function that promotes relaxation. The parasympathetic branch innervates its target organs including the heart *via* the vagus nerve. Vagal action occurs immediately, decreasing oxygen consumption, respiratory rate, blood pressure, and heart rate, leading to an increase in well-being, also known as the “relaxation response” (Benson, 1983; Shaffer et al., 2014). The parasympathetic response, which brings the body into balance through its responses to external stressors, is an important component of flow.

In order to measure flow at the physiological level, we turn to HRV. HRV is generated by the variation in consecutive heartbeat intervals. Changes in HRV result from the interaction between the sympathetic and parasympathetic arms of the autonomic nervous system and are modulated with, for example, changing environmental demands (Task Force, 1996; Pieper et al., 2010). A high HRV reading, that is greater variation in inter-beat intervals, indicates that the body has self-regulated itself by initiating a parasympathetic response. Since the flow state is found at the point where the parasympathetic system is activated to an extent that brings the body into balance with the sympathetic system. HRV analysis can be of help in the analysis of states of flow and other psychophysiological conditions.

A number of different systems regulate heart rate. These systems differ in speed (i.e., cycle time), and commonly three speed intervals – or frequency bands – are studied: the vagally mediated signals, where breathing is the main contributor, have the shortest cycle time of about 2.5–6.7 s, corresponding to the high frequency (HF) band (0.15–0.4 Hz). The low frequency (LF) band (0.04–0.15 Hz), corresponds to a cycle time of 6.7–25.0 s, and is most often claimed to be a marker of sympathetic tone and closely associated with blood pressure regulation. The longest cycle (>25 s) is referred to as very low frequency (VLF, <0.04 Hz) and seems to reflect thermoregulatory and baroreceptor systems (Kitney, 1980; Eckberg and Kuusela, 2005) and renin-angiotensin systems (Bonaduce et al., 1994) and also depends on physical activity (Bernardi et al., 1996) and parasympathetic outflow (Taylor, 1998). In HRV analysis, the signal strength (power) reflecting activity in the regulatory system corresponding to each of these three frequency bands is measured and expressed in milliseconds squared (ms^2^; Task Force, 1996). Total power (TP) indicates the synthesized overall activity for all three frequency bands. HF in heart rate is mainly an indicator of vagal (parasympathic) activity.

The state of flow has been investigated in musicians with different levels of expertise and in a variety of contexts, such as during live performance auditions and musical jam sessions (Wrigley and Emmerson, 2013; Hart and Di Blasi, 2015), as well as in experimental settings (de Manzano et al., 2010). Further studies also confirm that moderate sympathetic arousal and a co-activation of both main branches of the autonomic nervous system characterize task-related flow-experience (Peifer et al., 2014; Harmat et al., 2015; Tozman et al., 2015).

Additionally, Sawyer (2006) described the existence of group flow during musical collaboration and defined it as a “collective state of mind” when the performers were in “interactional synchrony” (Sawyer, 2006, 2007). However, HRV data represents a novel approach to measuring the experience of flow in musicians by means of physiological assessment. In the scientific literature, individual EEG recordings have been used in combination with video data to understand the emotional interplay involved in music therapy (Fachner et al., 2019), but there has been little research about flow and its mechanisms in interactive situations such as in performances by groups of musicians (Csikszentmihalyi, 1990; Sawyer, 2007; Hart and Di Blasi, 2015; Magyaródi and Oláh, 2015, 2017).

Two sets of qualitative data for characterization of the psychological states of the participants were collected.

Accordingly, the purpose of this exploratory study is to investigate how the quantitative data from HRV recordings can be combined with qualitative data in the study of flow. Our research question is therefore: can the combination of physiological and psychological responses in musicians help us understand individual and group state flow?

### A Matrix for Characterization of Demands in the Music

There was a need for a tool with which we could analyze the composition from the point of view of demands that the music makes on the musicians. We are not aware of any such measurement tools therefore a matrix was constructed for the characterization of three types of demands from the composer in the music for each one of the four musicians (violin 1 = V1, violin 2 = V2, viola and cello) and for each movement. It was a way of objectifying the music from a technical performance perspective. This offered a way to hypothesize how the individual musician could be expected to react to the playing of different parts of the quartet and relate that to possible flow-related experiences.

For each type of demand a four-graded scale was used (1 = lowest and 4 = highest demands). The three types of demands were:

#### Pitch

For string instruments, the general rule is that the higher the pitch, the more demanding the notes. This gets particularly difficult from the seventh position on the violin. In this quartet, there are requirements for the first violin in all the movements to play in positions above the seventh position, in the third movement up to the 10th position. Scores were defined in the following way in the matrix: 4 = At least seventh position, 3 = At least fourth–sixth position, 2 = At least second to third positions, and 1 = First position only.

#### Rhythm

Fast rhythm is always more demanding than slow. Scores: 4 = Several bars following one another with sixteenths in allegro-presto or 30-s in at least andante, 3 = Same as 4 but only occasional bars, 2 = Eighths in allegro-presto or sixteenths in at least andante, and 1 = Slower tempi.

#### Emotional Engagement

Scores: emotional engagement means that an emotionally charged theme has to be played in such a way that the audience is emotionally moved. 4 = During an entire movement, 3 = During most of the movement, 2 = During less than half of the movement, and 1 = Not at all.

The scores 1–4 estimated for all parts (V1, V2, viola, and cello) and all four movements in this particular quartet are presented in Table 1. The table shows that the first violin part is more demanding than the other parts (as in many of Haydn’s string quartets). The first violin has high demands for pitch, particularly in the first three movements. Fast rhythm adds to the technical demands in the first, second, and fourth movements. The contrast between the four instrumental parts becomes particularly evident in the second movement in which the first violin plays long sequences of 30-s in an emotionally moving melody. The viola also plays a similar passage of 30-s, but it only lasts for a few bars. In fact, the second movement has the form of a movement in a short violin concert in which the other instruments only accompany the first violin – with the exception of the short viola section. This also increases the emotional engagement demands on the first violin while the other instrumentalists should serve as support.

**Table 1:** This matrix presents the characterization of three types of demands in the music (pitch, rhythm, and emotional engagement) for each one of the four musicians (violin 1 = V1, violin 2 = V2, viola and cello) and for each movement.

	I	II	III	IV
**Pitch**				
V1	4	4	4	3
V2	2	2	2	2
Viola	2	2	2	2
Cello	2	2	2	2
**Rhythm**				
V1	4	4	3	4
V2	2	2	2	3
Viola	2	3	2	3
Cello	3	2	2	3
**Emotional engagement**				
V1	2	4	2	2
V2	2	3	2	2
Viola	2	3	2	2
Cello	2	3	2	2

For each type of demand a four-graded scale was used (1 = Least demanding and 4 = Most demanding).

Based upon the demands in the music the following prediction can be made:

1. V1 should have higher heart rate and lower HF (= high frequency power) in the HRV) than the others throughout
2. The second movement should, compared to the other movements, be associated with lower heart rate and higher HF in all musicians except in the V1 player due to the technical demands in that part. The viola player also has a technically demanding part during a short section of this movement, so the lowest heart rate and highest HF is expected to occur in the V2 and cello players. These two parts are mainly supporting the first violin in this movement.
3. During the second concert, when the music was repeated and the musicians were accustomed to this secondary school class audience, a lowered heart rate, corresponding to a lowered sympathetic activity, and an elevated HF, corresponding to increased parasympathetic activity, is expected.

The matrix provided us with a possibility to relate expected performance demands to flow-related experiences.

## Materials and Methods

### Participants

For this methodological experiment, the participants were all healthy chamber music students: two women, 25 and 29 years old, and two men 23 and 24 years old. They were enrolled at the time of the study at the Royal College of Music in Stockholm, Sweden. The musicians were participating in an advanced program, *Musethica*, which provides multiple opportunities for the students to play chamber music to lay audiences (see Theorell and Bojner Horwitz, 2019). The participants were recruited by direct inquiry.

None of the musicians were on medication of relevance. They were all young healthy adults, two men and two women.

### Context of the Data Collection

The musicians performed the same string quartet (Haydn’s string quartet 76, No 2 D-minor) to two different audiences of secondary school classes. The quartet has four movements and was unfamiliar to the audience.

The second movement of the piece of music performed in this study differs from the other movements in its character and composition: while the tempo is a relatively calm andante, the first violin plays a demanding segment of 30-S, incorporating high notes. This is quite technically difficult to perform. The difficult bars are interspersed with less demanding parts. Apart from the viola player, who also has some challenging rapid bars to play toward the end of the movement, the other instrumentalists have comfortable parts to play.

### ECG Recordings

Heart rate variability (HRV) was measured using a combined heart rate and movement sensor (Actiheart, Cambridge Neurotechnology, Ltd., Papworth, UK) that was able to record inter-beat intervals with good reliability and validity (Brage et al., 2005).^1^ This sensor was a small portable rechargeable unit (Actiheart) applied to the chest with temporary adhesive. The sensor recorded both heart rate and HRV. Since the LF power of the HRV is influenced by both the sympathetic and parasympathetic systems, we decided to focus our analysis on heart rate, mirroring the physiological arousal that is largely governed by the sympathetic nervous system, and in HF, correspondingly mirroring the parasympathetic system activity.

In the present study, measurement and analysis comprise HRV and heart rate.

The Actiheart recorder was fixed to the participants’ upper chest of the musicians by clips that fit standard ECG electrodes. The Actiheart recorder was firmly fixed to an electrode placed just below the apex of sternum while the wire running from the monitor was fixed to an electrode placed on the same horizontal level and as lateral as possible. In the Actiheart recorder, the analog signal was band-pass filtered (10–35 Hz), sampled with a frequency of 128 Hz, and processed by a real-time QRS-detection algorithm (Actiheart User Manual, 2010). During the recording, interpolated RR intervals with a resolution of 1 ms were stored in the memory. In the recording mode employed in the present study, the raw ECG signal is not stored by the Actiheart recorder (Kristiansen et al., 2011). The HF (0.15–0.4 Hz) and LF (0.04–0.15 Hz) were calculated by Actiheart 4 Software based on the recorded RR intervals. Missed beats in the IBI data were corrected according to the Actiheart User Manual (2010).

### The State Flow Scale

In previous studies (de Manzano et al., 2010; Harmat et al., 2015), flow has been measured using a subset of nine items from the FSS-2 (Jackson and Eklund, 2004) to assess flow experience within a particular event. Good psychometric properties of the FSS-2, as well as of the shorter English nine-item version of the test, similar to the one employed here, have been used in several studies with different samples (Jackson and Eklund, 2002, 2004; Jackson et al., 2008; Kawabata et al., 2008). In the Jackson and Eklund (2002) study, item identification sample reliability estimates for the FSS-2 ranged from 0.80 to 0.90, with a mean alpha of 0.85. Items are formulated as statements about subjective experiences of a performance (e.g., “I had feeling of total control.”), in response to which the respondent should agree or disagree. Answers are given on a Likert scale with nine steps ranging from 1 (strongly disagree) to 9 (strongly agree).

In our study, we used four of the nine questions that related to the state flow experiences (Csikszentmihalyi, 1990; Jackson and Eklund, 2004). These were challenge-skills balance, unambiguous feedback, feeling of control, and autotelic experience. This selection was justified based upon psychometric analysis conducted in a previous study (De Manzano et al., 2010) and informed by a judgment that it would be most conducive to the study to expose the instrumentalists to a minimum of questions during the intermissions between movements. Measurements were taken between each movement and during both public music performances.

### Flow Synchronization Scale

Interpersonal synchrony may increase prosocial behavior in which we become likely to trust and cooperate with one another (Cirelli et al., 2014). According to the Flow Synchronization Theory (Magyaródi and Oláh, 2015, 2017), flow synchronization is a psychological mechanism stimulating the group members to interact with each other, and to work for common goals in cooperation during an optimally challenging situation. Thus, interaction includes experience of cooperating as well as iterative exchange of initiatives, ideas, and views. People who experience this know their exact purpose and share a common strategy with others to achieve this goal. Partners help each other, integrate with one another consistently, motivate themselves, and learn from each other. Looking back on the experience, they realize how much they have developed during the activity and how they positively affected each other’s performance. Group members also display a sharing of their knowledge, giving feedback to one another. This may support emergent motivation to engage in ongoing collaboration (Csikszentmihalyi and Nakamura, 2010). In order to study how experience during an interactive situation becomes optimized through such synchronization we used the Flow Synchronization Questionnaire (FSyQ) which was developed by a Hungarian research group at Eötvös Lórand University, Budapest (Magyaródi and Oláh, 2015). It enabled us to measure the perceived quality of social interactions during public music performances.

The development of the FSyQ measure was based on both the rational and empirical test establishment traditions. The questionnaire contains 28 items and 5 latent factors that focus on the motivational and coordination (task- and relationship-focus) aspects of the experience: (1) Synchronization and effective cooperation with the partner (12 items, *α* = 0.93); (2) Experience of engagement and concentration (five items, *α* = 0.83); (3) Motivation and positive impact on the partner (three items, *α* = 0.82); (4) Motivation and learning for the person (four items, *α* = 0.80); and (5) Coordination with the partner during the activity (four items, *α* = 0.81). The internal consistencies of the subscales are adequate and the Cronbach’s alpha reliability of the original questionnaire (total score) is *α* = 0.94 (Magyaródi and Oláh, 2015). In a recent article (Olsson and Harmat under review) presented the Swedish version of the questionnaire (Olsson and Harmat, Frontiers in Psychology, under review). The Swedish version was developed by first translating the original FSyQ from Hungarian into Swedish, using an independent translator and then back into Hungarian. After this, the original and the back translated FSyQ versions were compared, and a Swedish version was created. This procedure is according to the classic back-translation method developed by Brislins (1970, referred to in Cha et al., 2007). We tested the Swedish version of the questionnaire on a small sample (*n* = 62). The internal consistency of the questionnaire is adequate with a Cronbach’s *α* = 0.93. However, we assessed only seven items out of the 28 items of the questionnaire after each music performance based on the highest factor loads of each dimension, i.e., (1) Synchronization and effective cooperation with the partner (1); (2) Experience of engagement and concentration (two items); (3) Motivation of and positive impact on the partner (one item); (4) Motivation and learning for the person (one item); (5) Coordination with the partner during the activity (two items).

### Qualitative Interview

A single semi-structured group interview (duration of 1 h) was conducted at the Royal College of Music in Stockholm with the four musicians after the culmination of both of their performances. The interview was recorded, transcribed and analyzed using a phenomenological hermeneutic method. The data takes the form of the transcribed participant discussion, sitting around a table together and sharing answers to the researcher’s questions. Focus group questions probed how musicians evaluated their experiences of interaction with the audience and their experiences of each musical movement. We asked open questions to achieve this, such as: describe how you remember the concert?, How did you perceive the interplay among you?, Are there special moments you remember from the concert?, and Did you feel any flow during the concert?

The data were processed through developing a Naïve Reading – a summary of the data derived from detailed descriptions of participants’ experiences – in line with our research aim (Bojner Horwitz et al., 2003, 2016; Lindseth and Norberg, 2004; Grape Viding et al., 2017). To conduct a Naïve Reading, the researcher reads the focus-group transcripts several times in order to recognize and synthesize the essence of the informants’ discussion. The analytical prism of the research question (How do musicians’ physiological and psychological responses indicate individual and group state flow?) was employed to delineate and distinguish relevant “meaning units” contained in the accounts of musicians’ experiences. From this, we gained insights into the interplay among musicians that served to strengthen the physiological findings.

### Ethical Considerations

Ethical approval was obtained for the study (Dnr 2017/1009-31/1 Central Ethical Review Board in Stockholm, Sweden, and both oral and written informed consent was obtained from the participants.

## Results

The results are presented in the following order: the focus group interview, the state flow scale, the flow state and synchronization scales, ECG recordings.

### The Focus Group Interview

The musicians’ experiences of interacting with the audience, reading their responses to the performance, and their perceived interactions with other musicians in the ensemble are summarized in a Naïve Reading. The Naïve Reading is a synthesis derived from the following quotations from the group interview:

“The second movement, with intonation: thought it was difficult. We practiced it pretty much.”

“Each time there were the same difficulties. We worked a lot with intonation in the second movement. It became so clear that we needed to support each other a lot.”

“The second movement is quite slow. Playing slow is more difficult to do in front of a school audience. They have not chosen to be an audience. I have to make an extra effort, to make them appreciate it.”

“It might just be so in my head … but the school class may not think it is as cool with slow and calm tempo as in other audiences.”

“I have a solo in the second movement and you are helping me (to play), and then you listen more to me then, ‘maybe you would become even more compassionate if I made big mistakes, then probably your compassion would increase because of that’… Probably, your compassion will then increase because of that.”

“Empathy in the second movement. The music is beautiful and captivating in some way and for them (the other musicians) to be able to share a good interplay, so you have to listen to me playing the melody. There is empathy.”

“I felt the most of interaction in the second (movement)…”

“The interaction was best for me in the first movement, and maybe also in the second one. Maybe not right from the start (first time performance) but when we played it over again (the second time). I think we had practiced the second (movement) the most.”

“Felt like we were breathing together in the second (movement).”

These quotes regarding flow-related experiences provide a triangulation point for the physiological data reported below.

Moreover, the musicians seem to share some elements of their experiences in the second slow movement. Deriving from the Naïve Reading, the findings can be summarized as follows:

The second movement was the most difficult part of the whole piece and the musicians had practiced the second movement the most. They had also worked a lot with intonation in the second movement. Some of the observations the musicians made about the second movement were potentially related to the experience of group flow: they expressed the need to support each other because of the problems they had with intonation during practice of this movement. They felt that they were, as musicians, breathing together in the second movement.

The participants were also asked to outline the different elements of their experience that contributed to the dynamic of their interplay as musicians:

- External circumstances (time of day, early morning was difficult).
- Supporting arrangements (in this case, good teachers in the classroom).
- Distracting factors (our ECG recordings and filling out forms).
- The ensemble’s beliefs about audience attitude (perhaps being too negative in this case).
- The attitude of the four musicians toward one another.
- The attitudes of the audience/audience acquaintance with the kind of ensemble and the music.
- The content and difficulty of the music.
- Individual preparations (practiced a lot/a little on the piece).
- Group exercise (questioning whether they had practiced for intonation sufficiently).

This synthesis was derived from quotations in the group interview, such as:

“We need to support the soloist.”

“We have practiced a lot, especially the second movement.”

“But here I did not feel that they (the audience) loved the second movement (in class).”

“It took some time to get used to the ECG equipment.”

“I think we have found a compassionate attitude toward another.”

### Individual State Flow and Group Flow Synchronization

**Figure 1:**
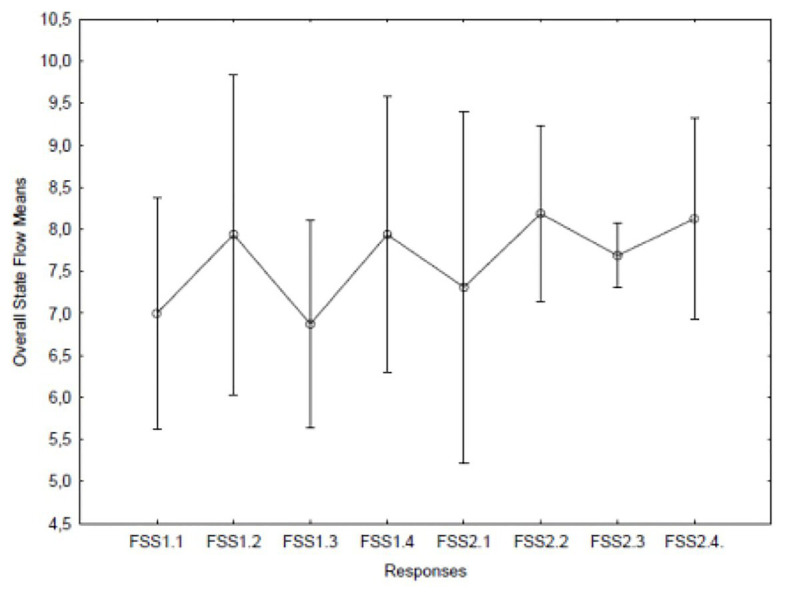
Flow means for the first concert with movements 1–4 (1.1–1.4) and correspondingly for the second concert with movements 1–4 (2.1–2.4). The average flow had its highest value after the second movement on the second occasion. The vertical lines are 95% CIs.

One-way ANOVA was performed in order to measure the significance of the variation in flow scores over time during the different movements and performances. Individual flow assessments taken after each movement of the performance did not show any significant variation over time (Figure 1).

We collected data from the FSyQ after the first and the second performances (Magyaródi and Oláh, 2015), in order to examine the psychological mechanisms involved when the group members were interacting with one another, during an optimally challenging situation. We did not find significant differences between the two performances with regard to flow synchronization. A paired sample t-test indicated that there was no statistically significant difference between the two performances with regard to flow synchronization.

### ECG Recordings of Heart Rate and HRV

**Figure 2:**
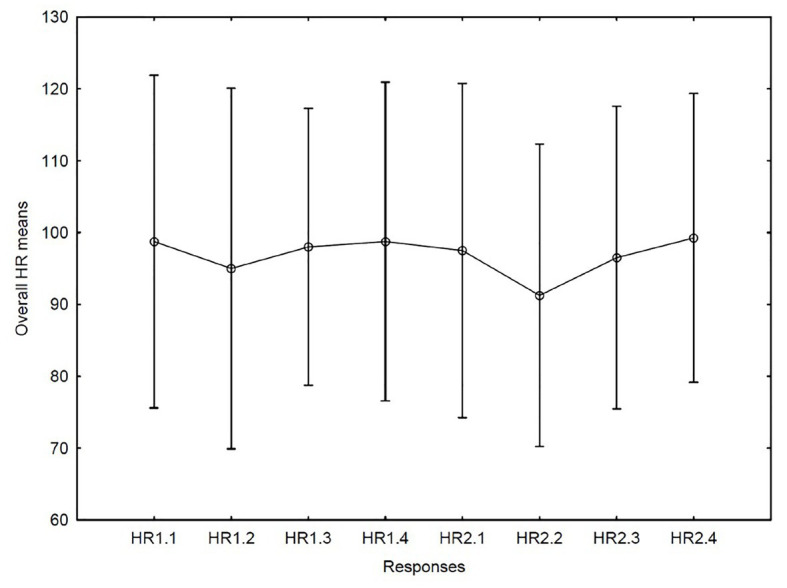
Heart rate (HR) means for the first concert with movements 1–4 (1.1–1.4) and correspondingly for the second concert with movements 1–4 (2.1–2.4).

Electrocardiogram (ECG) recordings were subjected to analysis of heart rate and HRV. The four movements in the string quartet on the two occasions (2*4) were analyzed as distinct periods. It was observed that the mean heart rate in the four musicians was lowest when they played the second movement on the second occasion. Using one-way ANOVA, variation over time was not significant (*F* = 2.15, *df* = 1/7, *p* = 0.08, see Figure 2).

**Figure 3:**
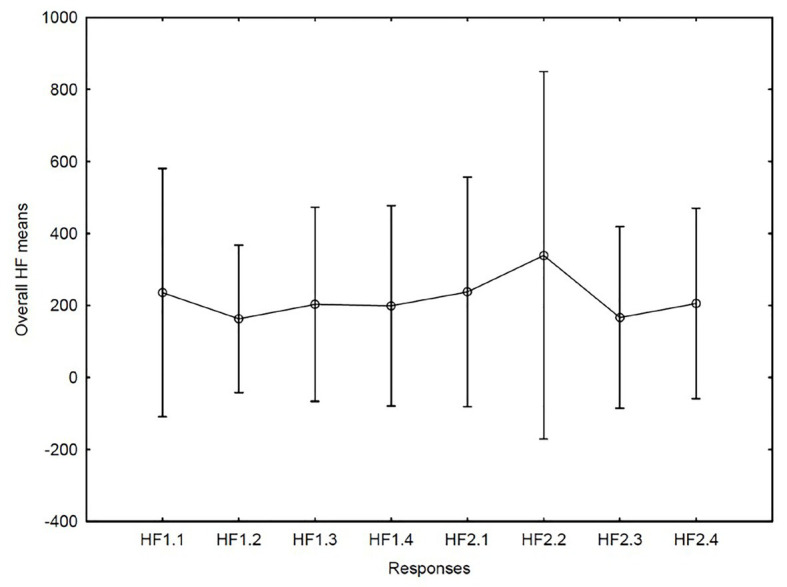
High frequency (HF) means for the first concert with movements 1–4 (1.1–1.4) and correspondingly for the second concert with movements 1–4 (2.1–2.4).

**Figure 4:**
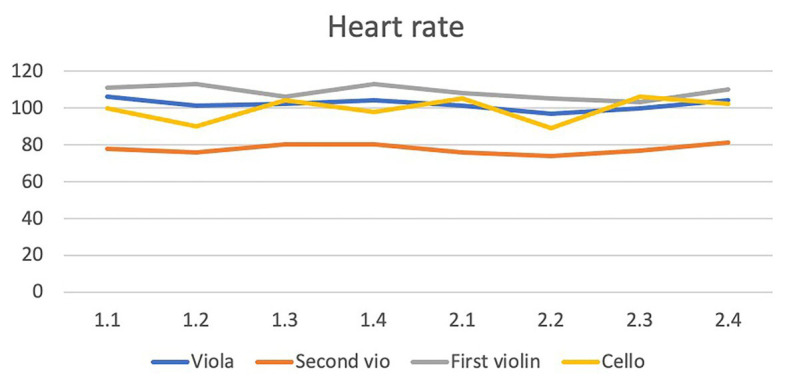
The figure shows that the first violinist (gray line) had a relatively high heart rate (103–111 beats per minute) throughout both concerts (1.1–2.4) and when the second movement was performed in both concerts (1.2 and 2.2); with 113 beats per minute during the first concert’s second movement (1.2) and 105 beats per minute during the second concert’s second movement (2.2). The second violinist (orange line), and the cellist (yellow line) have their lowest heart rates during the second movement on both occasions.

**Figure 5:**
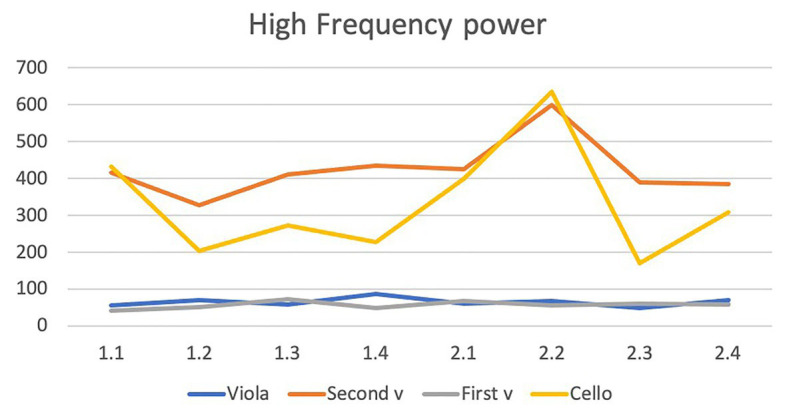
The figure shows that the first violinist (gray line) had the lowest HF power during both concerts and during the second movement (2.2). From the first (1.1–1.4) to the second (2.1–2.4) concert, a dramatic increase in HF occurred in the second movement (2.2) in both the cellist (yellow line) and the second violinist (orange line). The viola player (blue line) shows data similar to the first violinist’s data (gray line), and none of them have any increase in HF power during the second performance’s second movement.

HRV: no differences between the occasions or movements were discovered with regard to variations in LF power of the HRV (see Figure 2). For the HF power (which is reportedly the parameter that mirrors parasympathic activity) the highest mean was found in the second movement on the second occasion. We also analyzed the LF power of HRV but found no systematic changes. Variation over time did not reach statistical significance (*F* = 2.00, *df* = 1/7, *p* = 0.10, (see Figure 3). Each one of the four musicians’ mean heart rates and high frequency means are illustrated separately in Figures 4, 5.

Comparing with our expectations (see above), we found that:

Prediction 1: Yes, the first violin player had the highest heart rate. The V1 player’s mean heart rate during the four movements in the two concerts ranged from 103 to 113. The corresponding means for V2 were 74–81, for the viola player 97–106 and for the cello player 90–106. With regard to HF, the V1 player had low HF. However, an equally low HF was observed in the viola player.
Prediction 2: Yes, in general, the musicians had a lower heart rate during the second movement than in the other movements both during the first and the second concert. The exception was the V1 player who did not show a lowered heart rate during this movement. For HF, the prediction was correct for the V2 and cello players but only during the second concert.
Prediction 3: The V1 and the viola player had lower heart rates during the second concert than during the first one, but for the other players there was no clear difference between the two concerts. During the first concert the mean HR during movements varied between 106 and 113 in the V1 player, between 76 and 80 in the V2 player, between 101 and 106 in the viola player, and between 90 and 104 in the cello player. The corresponding numbers during the second concert was 103–110, 74–81, 97–104, and 89–106.
HF: In the V1 and viola players, no clear changes were observed between the two concerts. These two players both had low HF. The two other players (V2 and cello) had much higher HF than the other players throughout, and a pronounced increase took place from the first to the second concert during this movement (327–598 in the V2 player and 204–635 in the cello player), which indicates a stimulated parasympathic system.

## Discussion

In this case study, we used a combined methodology mirroring aspects of possible state flow in four musicians: perceived experience of synchronized social interactions and physiological states in the performance. By conducting recordings performed on a limited number of players, we have been able to explore a novel methodology that combines psychological and physiological data in order to characterize different aspects of flow.

The *Musethica* program with its repetition of concerts, under similar conditions, provided an opportunity for us to study behavioral and physiological parameters on two different occasions consecutively. It is important to remember in interpreting the value of this experiment and its potential usage on other studies, that there are of course physiological and psychological external factors not related to the playing itself that could have influenced our assessments. The findings on HR and HF only partly confirmed our predictions. Despite this, we were able to confirm predictions that the first violin player should have higher heart rate than the others and that the two players with a supportive role during second movement had low HR and high HF during the second concert when the group felt “safer.” Despite our small sample size, the total between movement variation in heart rate and high frequency power approached significance. That the LF power did not show systematic changes may be due to the fact the LF power of HRV is influenced concomitantly by the sympathetic and the parasympathetic system (Porges, 2007).

Are the results of this study consistent with what one would expect from the musicians’ experiences? Do they mirror the four elements of the performance outlined in the matrix constructed at the outset of the research? If we consider the patterns of the state flow response in the data, we do in fact see the same patterns during the two performances: the first and third movements obtained lower flow ratings compared to the second and the fourth movements in both performances. The mean ratings of the second performance were slightly higher than the fourth. In addition, the second time the slow movement was played the very lowest mean heart rate was recorded. Similarly, the highest mean high frequency power was found in the second movement but only during the second performance. This may indicate that the musicians experienced a physiologically more relaxed state during the performance of this movement on the second occasion. High HF and low HR also coincided with the peak mean flow rating.

Furthermore, the ECG analysis showed that the first violinist had the highest heart rate and the lowest HF during the second movement of the performance. However, this violinist also showed clearly decreased heart rate from the second movement in the first concert to the second movement in the second concert (113 vs. 105 beats per min). The most pronounced decrease in heart rate from the first to the second movement occurred in the two musicians with the least technically demanding parts.

These findings are consistent with the expectation, based upon the matrix scores (see above) that V1 should have higher heart rate and lower HF than the others throughout. They are also in line with our hypothesis that the second movement should, compared to the other movements, be associated with lower heart rate and higher HF except in the V1 player due to the technical demands in that part and also in the viola player during a short section of this movement. Accordingly, the lowest heart rate and highest HF were expected and also occurred in the V2 and cello players.

In addition, in keeping with our hypothesis, during the second concert when the music was repeated and the musicians were accustomed to this secondary school class audience, on average a lowered heart rate and an elevated HF, corresponding to increased parasympathetic activity, was found. The increase in parasympathetic activity was pronounced in the V2 and cello players in the second movement during that concert. Difference in the musical content cannot explain the differences between the patterns observed in the two concerts, and thus we must consider contextual factors, such as their playing the same music the second time during the same day; familiarization with the setting; a better general atmosphere in the room, and connection with the audience; or indeed a better overall sense of mastery during the second performance. Taken together these findings are consistent with our predictions and indeed de Manzano’s and colleagues’ previous work (2010) in which we used piano playing as a flow-inducing behavior in order to analyze the relationship between subjective flow reports and psychophysiological measures.

However, the results based upon the questionnaire in the present study contrasted with the study with professional pianists. This finding related to autotelic experience of each movement, the enjoyment of the task among musicians. In the previous study by de Manzano et al. (2010), in which professional pianists performed a difficult self-selected piece on five different occasions, the occasion with the highest flow score (measured according to Csikszentmihalyi) was compared with the other occasions. It was shown that the activity in the laughing muscle Zygomaticus major (assessed by means of continuous EMG recordings) was higher during the occasion with the highest flow rating than during the other occasions. The participants also displayed a deep slow breathing pattern when the mean flow score was high. What was surprising is that these musicians also showed a higher heart rate on that occasion than on the other four occasions. This was interpreted as demonstrative of flow because it was an example of combined sympathetic and parasympathetic activation; a state a high arousal manifested in a high heart rate, combined with a state of relaxation manifested in deep slow breathing stimulating the vagus nerve. According to the interpretations of these researchers, the findings of our study during the second movement would be more indicative of a relaxed happiness state.

Our qualitative data illustrating musicians’ “experiences” of flow, suggested that they had a strong shared interpretation of the feeling of the music, its demands, and practical approaches to these features. This has been missing from previous studies and gives us greater insight into how the physiological measurements correlated with a potential flow state even when the demands on the players may be moderately unbalanced (Peifer et al., 2014).

### Implementation and Future Development of the Methodology

In our study, the recordings were made in real life conditions and there was only minimal time for baseline recording since the electrodes were applied immediately before the concert. In implementing this methodology in the future, it would be useful to establish robust baseline data. This would mean that more rigorous analysis in relation to baseline data could be performed. Sampling of ECG data was, however, performed continuously during the concerts and each movement was treated as a separate period. The Actiheart technology was able to identify movements in the same way as during walking and “normal” activities of daily life. We would recommend that measurements are taken continuously rather than at sampled intervals for accuracy. Regarding emotional “standard” ratings, i.e., our matrix for characterization of demands in the music, this assessment instrument will need systematic psychometric evaluation in the future.

We experienced some technical difficulties: the type of instrument played by each musician did not seem to influence the data overall; however, one of the instrumentalists had a problem with a lost electrode during the last part of one movement. A small segment of the ECG was lost as a result and the electrode was re-applied during the subsequent intermission. However, in general, the equipment functioned well, and the musicians felt comfortable with it during their performances. Furthermore, the elements of the Actiheart that measured muscular movements (although not reported on here) did not appear to interfere with the electrocardiographic analyses. Any ectopic beats in the cardiac rhythm, which are common even among perfectly healthy young people, were identified by the automated program and did not affect analysis.

In terms of future subjects for research, firstly, one could use this methodology as a basis for a study of physiological synchronization during flow. This would require specialist ECG analytical skills. We did not have access to this expertise. Secondly, the audience constitutes an important part of the interplay that occurs during a musical event and offers avenues for future investigation. In relation to this, one might consider for example the type of music performance and its impact on the dynamic interaction between musicians and audience. In our study, the musicians performed a classical piece by Joseph Haydn that did not require any musical improvisation (Hart and Di Blasi, 2015). According to Keeler et al. (2015), there were no differences in the intensity of the flow experience when a small group of singers performed pre-composed pieces or improvised together. However, there is a question how the different traditions and different types of musical events/genre shape the interactions between musicians and their audiences. For example, maybe jazz musicians perceive more concrete feedback from their audiences during the performance (e.g., hand clapping after an improvised moment) than musicians during other types of musical events such as classical music performances (Brand et al., 2012). However, silence can also be a positive feedback from the audience during a classical music performance in accordance with genre convention. Future research is needed to clarify the different types of musical events/genres. It is known from extant research that positive feedback and public acknowledgment may not only enhance performers’ self-confidence and motivation (LeBlanc et al., 1997; O’Grady 2009; Baker and MacDonald, 2013), but also facilitate strong interplay between audiences and performers. Vice-versa too, when musicians are strongly engaged and have a feeling of mastery (ease-less effort or “flow”; de Manzano et al., 2010; Harmat et al., 2011, in press) their emotional state is also likely to have a strong emotional influence on the audience. This suggests a mutual relationship between musicians and their audiences that may be shaped by the specific nature of the performed piece.

## Conclusion

The combination of physiological data *via* ECG recordings, and psychological data *via* expressive narratives provided new insights and has contributed to a proposal of a novel methodology. Although the number of observations was limited in this pilot study, we can conclude that the proposed methods are indeed feasible to investigate the interplay between chamber musicians and may provide meaningful results in further studies. The qualitative data illustrating the musicians’ “experiences” of flow, suggested that they had a strong shared interpretation of the feeling of the music, its demands, and practical approaches to these features. This has been missing from previous studies and gives us greater insight into how the physiological measurements correlated with a potential flow state even when the demands on the players may be moderately unbalanced. Our study indicates that it is possible to use these kinds of mixed-methodology techniques to examine perceived flow experiences at individual and group levels during musical performances.

## Data Availability Statement

The raw data supporting the conclusions of this article will be made available by the authors, without undue reservation.

## Ethics Statement

The studies involving human participants were reviewed and approved by Dnr 2017/1009-31/1 Central Ethical Review Board in Stockholm, Sweden. The patients/participants provided their written informed consent to participate in this study.

## Author Contributions

EH: ethical approval, project administration, conceptualization, writing, formal quant analyses, and editing. LH: data curation, methodological consideration, formal quant analyses, and writing. WO: conceptualization, supervision, writing, editing, and project administration. TT: ethical approval, supervision, writing, editing, methodological consideration, and formal analyses. All authors contributed to the article and approved the submitted version.

### Conflict of Interest

The authors declare that the research was conducted in the absence of any commercial or financial relationships that could be construed as a potential conflict of interest.

## Footnotes

^1^The movement measurements that were recorded do not form part of the data for this paper.
